# Diabetic Retinopathy, lncRNAs, and Inflammation: A Dynamic, Interconnected Network

**DOI:** 10.3390/jcm8071033

**Published:** 2019-07-14

**Authors:** Saumik Biswas, Marie Sarabusky, Subrata Chakrabarti

**Affiliations:** 1 Department of Pathology and Laboratory Medicine, Western University, London, ON N6A5A5, Canada; 2 Department of Optometry and Vision Science, University of Waterloo, Waterloo, ON N2L3G1, Canada

**Keywords:** diabetic retinopathy, inflammation, lncRNAs, epigenetics, histone modifications, DNA methylation, miRNAs

## Abstract

Diabetic retinopathy (DR) is reaching epidemic levels globally due to the increase in prevalence of diabetes mellitus (DM). DR also has detrimental effects to quality of life, as it is the leading cause of blindness in the working-age population and the most common cause of vision loss in individuals with DM. Over several decades, many studies have recognized the role of inflammation in the development and progression of DR; however, in recent years, accumulating evidence has also suggested that non-coding RNAs, especially long non-coding (lncRNAs), are aberrantly expressed in diabetes and may play a putative role in the development and progression of DR through the modulation of gene expression at the transcriptional, post-transcriptional, or epigenetic level. In this review, we will first highlight some of the key inflammatory mediators and transcription factors involved in DR, and we will then introduce the critical roles of lncRNAs in DR and inflammation. Following this, we will discuss the implications of lncRNAs in other epigenetic mechanisms that may also contribute to the progression of inflammation in DR.

## 1. Introduction

The increase in the global prevalence of diabetic retinopathy is intimately connected to the soaring prevalence of diabetes mellitus (DM) to an epidemic proportion [1,2,3,4,5,6]. Diabetic retinopathy (DR) is the leading cause of blindness in the working-age population and the most common cause of vision loss in individuals with DM [7,8,9]. In 2015, 2.6 million people suffered from visual impairment due to DR; this figure is projected to reach 3.2 million people in 2020 [6,10]. As the life expectancy of individuals with diabetes increases due to medical advances, the prevalence of DR is expected to further magnify unless improvements are made in the current diagnosis, management, and treatment of this disease through understanding of the underlying pathogenetic mechanisms. Moreover, a persistent and complex problem surrounding the adherence to diabetic eye care guidelines among patients with diabetes due to the lack of knowledge of diabetic complications exist, as well as the asymptomatic features of the disease in the presence of major microvasculature changes, which likely perpetuates this increase in prevalence [11,12,13]. DR is a chronic microvascular complication of DM and is associated with a longer duration of diabetes and poor control of blood sugar, lipids, and blood pressure [14,15]. The majority of diabetics with type 1 and greater than 60% of diabetics with type 2 develop signs of DR within 20 years of diagnosis of DM [16,17,18,19,20,21]. DR progresses from non-proliferative diabetic retinopathy (NPDR) to more advanced forms of vision-threatening diabetic retinopathy that include proliferative diabetic retinopathy (PDR) and diabetic macular edema (DME). Clinically, NPDR is differentiated from more advanced forms of DR by the lack of neovascularization; however, an eye with NPDR may present with classic DR signs such as microaneurysms, intraretinal hemorrhages, venous beading, intraretinal microvascular abnormalities, and hard exudates [22]. PDR is characterized by the presence of retinal neovascularization due to ischemia that results from vascular occlusion [22]. DME occurs as a result of the accumulation of fluid in the neural retina, which leads to thickening of the retina and cystoid macular edema. DME is another important factor to be considered, as it may be present in any of the stages of DR [22]. Hyperglycemia is implicated in the pathogenesis of DR and induces a variety of biochemical pathways including genetic and epigenetic factors, advanced glycation end-products formation, polyol pathway, protein kinase C pathway, hexosamine pathway, retinal renin-angiotensin system, and numerous inflammatory mechanisms. With the prevalence of DR reaching paramount levels and the risk this disease poses to vision and thus quality of life, understanding the molecular mechanisms implicated in the pathogenesis of DR becomes critical. In this review, we explore the current state of knowledge surrounding the roles of long non-coding RNAs (lncRNAs) and inflammation in DR. Further, we will critically discuss the relevance of this knowledge to the pathogenesis of DR and the importance of novel approaches to diagnosis, treatment, and management of DR that go beyond the current standards of care.

## 2. Inflammation and Diabetic Retinopathy (DR)

Microvasculature instability in DR is a result of the combination of increased vascular permeability and vascular occlusion [22]. Vascular endothelial growth factor (VEGF) is a known contributor to vascular dysfunction in later stages of DR through increasing vascular leakage and angiogenesis [23,24]. Anti-VEGF therapy is useful for the treatment of DME as well as later stages of DR, when significant alterations (angiogenesis) to the retina have occurred. Moreover, anti-VEGF treatment has significant side-effects, requires repeated intraocular injections, and most importantly, only approximately 50% of patients respond to therapy [25]. There has been increasing evidence for the role of inflammation in the development and progression of DR; however, the detailed mechanisms initiating these inflammatory changes have yet to be elucidated [26,27]. Multiple inflammatory mediators and transcription factors work in concert to mediate such effects.

### 2.1. NF-κB

NF-κB is a ubiquitous transcription factor regulating the expression of cytokines, chemokines, growth factors, and cell adhesion. Most commonly, NF-κB is composed of p65 and p50 subunits, which when activated translocates into the nucleus as a p50-p65 heterodimer and initiates pro-inflammatory protein transcription (notably, iNOS2, ICAM-1, IL-1β, and TNF-α) [28,29,30,31,32,33,34,35,36,37]. Due to this, NF-κB is suggested to play a critical role in the development and progression of DR by inducing an overt-inflammatory response [31,33]. Hyperglycemic induced activation of NF-κB occurs very early in the development of DR and is an important signaling pathway that induces apoptosis in retinal endothelial cells [38,39]. Retinal capillaries of diabetic eye donors show increased numbers of retinal pericytes with activated NF-κB relative to non-diabetic donors, while endothelial cells in both were negative [40]. In addition, NF-κB activation induced by hyperglycemia may have pro-apoptotic consequences in retinal pericytes by accelerating loss of these cells in DR [40]. Interestingly, selective inhibition of NF-κB activation with dehydroxymethylepoxyquinomicin inhibited diabetes-induced retinal leukostasis and retinal expressions of ICAM-1 and VEGF in vivo [41]. Nevertheless, less specific therapies such as salicylates (aspirin, sodium salicylate, and sulfasalazine) have been shown to inhibit NF-κB activation in diabetes; thus, inhibiting degeneration of retinal capillaries and preventing ganglion cell death in diabetic rats [42]. In addition, non-specific therapy using multiple antioxidants including ascorbic acid, β-carotene, and selenium has been demonstrated to impede the development of DR through inhibition of NF-κB in diabetic rats [43].

### 2.2. Cytokines and Chemokines

A number of inflammatory cytokines and chemokines including IL-6, IL-8, IL-1β, and TNF-α have been shown to be elevated in diabetic vitreous samples [44,45,46]. Interestingly, one study showed higher concentrations of IL-8 and TNF-α in vitreous samples from eyes with NPDR than eyes with PDR [44]. However, another study demonstrated increasing concentrations of IL-1β, IL-2, IFN-γ, TNF-α, IL-4, IL-5, IL-6, and IL-10 in the aqueous humor associated with increasing severity of DR [47]. Similarly, in the context of DME, significant differences have been reported for several cytokines in the aqueous humor of DME patients and intravitreal administration of aflibercept (an anti-angiogenic agent) was shown to decrease the concentration of certain cytokines (including VEGF, IL-6, and IL-1β) [48]. Of note, the total vitreous protein concentration between patients with NPDR and PDR is comparable, which likely suggests that increased protein levels found in these samples is likely attributed to secretion rather than vascular leakage into the vitreous due to increased permeability [44]. These inflammatory mediators are produced by activated microglia, macroglia, endothelial cells, and even neurons at more advanced stages of DR [47]. Regardless, inflammatory cytokines are seen in early DR and the inflammatory response progresses throughout all cell types of the retina and mediate DR progression [44,47]. IL-1β is likely a crucial mediator associated with early damage in DR and the increasing concentration throughout the development of DR might promote the inflammatory process and initiate the production of other inflammatory mediators [49,50]. The accumulation of these inflammatory mediators has been proposed to contribute to angiogenesis and neurodegeneration in DR. Angiogenic responses of endothelial cells is induced directly by inflammatory cytokines such as IL-1β, IFN-γ, and TNF-α and indirectly by inducing endothelial cells to produce growth factors rather than a direct effect of hyperglycemia on endothelial cells [51,52,53,54]. Inflammatory cytokines also stimulate endothelial cell secretion of adhesion molecules such as ICAM-1; thus, promoting leukostasis [55]. Cumulatively, these processes lead to microvascular instability comprised of increased vascular permeability which allows increased leakage of vascular fluid and migration of immune cells into the retina as well as vascular occlusion due to endothelial cell degeneration [56,57,58,59]. The resulting ischemia and hypoxia of the retina promotes VEGF expression and further pro-inflammatory cytokine and chemokine production resulting in angiogenesis in PDR. VEGF is a double-edged sword in not only promoting inflammation and angiogenesis, but also promoting neuronal growth, differentiation, and survival [60,61,62]. Early in DR, cytokines and growth factor production may be a way to maintain neuronal function through increasing VEGF levels; however, as levels of proinflammatory mediators increase, they become detrimental and impair the positive effect of VEGF leading to eventual neuronal death in the retina.

### 2.3. Complement System

The complement system, a part of the innate immune system with a central role in host defense against infectious pathogens, has been shown to be dysregulated in DR. A fully activated complement pathway leads to C3/C5 convertase generation and, ultimately, to the formation of the membrane attack complex (MAC), which can kill pathogens, and in some instances, host cells; thus, potentially contributing to neurodegeneration in DR. Moreover, some studies suggest that dysregulation through elevated complement protein (C5b-9) deposition in the retinal vascular lumen and reduction of complement inhibitor proteins (CD55 and CD59) may be related to DR progression [63,64]. Additionally, hyperglycemic by-products, such as methylglyoxal greatly impair the function of complement regulatory proteins including the C1 inhibitor [65,66]. Deposition of C5b-9, the terminal product of the complement pathway, is present within the retinal blood vessels of diabetic patient donors, while notably not present in non-diabetic patient donors [65]. Another study found extensive deposition of C5b-9, C3d, and vitronectin (acts by forming a stable complex with extracellular C5b-9) in the retinal vascular lumen of patient donors with clinically evident DR, but the absence of the above in the majority of control patient donors [63]. Extensive deposition of complement factors in the retinal vascular lumen leads to MAC formation, which likely contributes to retinal endothelial cell death and increased retinal vascular permeability in diabetes [64]. Key players in the complement pathway (C4b, factor B, C3, and C9) are also elevated in the vitreous of patients with PDR relative to non-diabetic controls [67,68]. Complement pathway factors such as C3a and C5a are chemotactic and activate neutrophils, which parallels with findings from studies demonstrating increased numbers of neutrophils in diabetic retinal vessels [69]. Activated neutrophils worsen microvascular instability in DR via incurring damage to the endothelium, which leads to increased levels of plasma components in the connective tissue matrix, potentially exacerbating the inflammatory response found in diabetes [69]. Lastly, C5aR is constitutively expressed on Müller cells, the expression of which is up-regulated by prostaglandin E2, and most critically, hyperglycemia, and is associated with upregulation of IL-6 and VEGF leading to increased retinal endothelial cell proliferation and permeability [70].

Given the complex nature of cellular environments, the above summary of inflammatory processes involved in the development and progression of DR are by no means exhaustive. Though a detailed discussion of other DR-related molecular alterations (i.e., in matrix metalloproteinases [71], toll-like receptors [72], and α-crystallins [73]) goes beyond the scope of this review, future research is expected to provide additional knowledge on the inflammatory pathways contributing to DR. Considering the impact inflammatory pathways have in the pathogenesis of DR, inhibition of these inflammatory processes may be an appealing option to integrate into the future standard of care. However, further understanding on the particular initiators propagating inflammation in DR is needed. In the last few years, accumulating evidence has suggested that non-coding RNAs, especially lncRNAs may be aberrantly expressed in diabetes and may play a putative role in the development and progression of DR through modulation of gene expression at the transcriptional, post-transcriptional, or epigenetic level [74]. In addition, the role of lncRNAs in DR deserves to be investigated as they may also have a role as new biomarkers offering diagnostic value or in future novel treatments of DR. Thus, in the next section(s), we will explore the role of lncRNAs in the pathogenesis of DR and the crucial nature of their role in stability and maintenance of gene expression patterns, especially relating to inflammatory pathways.

## 3. LncRNAs: Novel, Emerging, Regulatory RNA Molecules

As novel sequencing technologies continue to rapidly emerge [75], the identification of non-coding loci, in parallel, grows at an unprecedented rate. Non-coding DNA regions constitute more than 98% of the human genome [76] and due to the pervasiveness of transcription [77], the transcriptional products from certain non-coding RNA genes can serve critical roles in a diverse array of biological processes, ranging from embryonic development [78] to proper maintenance of the immune system [79]. Amongst the various non-coding RNAs, long non-coding RNAs (lncRNAs) are a class of fundamental RNA transcripts that are larger than 200 base-pairs and generally do not have protein-coding potential. Mechanistically, lncRNAs are capable of governing gene expressions through a number of different means: (i) Serving as a decoy for transcription factors, which can enable gene inactivation [80], (ii) guiding certain proteins, such as chromatin-modifying enzymes, to certain regions of the DNA [81], (iii) acting as a scaffold for the assembly of multiple protein subunits into complexes [82], (iv) functioning as a molecular sponge that sequesters pertinent microRNAs (miRNAs) to allow or prevent the translation of distinct messenger RNAs (mRNAs) [83,84], or by (v) directly enhancing the activation of neighboring genes [85]. Given the complex regulatory nature of these transcripts, certain lncRNAs may follow more than one of the above mechanisms and present with distinct functionalities and structural features depending on their subcellular localization. For example, nuclear-retained lncRNAs are typically implicated in transcriptional regulation [86], alternative splicing [87], and in the organization of nuclear architecture [88]; while, cytoplasmic lncRNAs are involved in post-transcriptional modifications that determine the stability and translation potential of mRNAs [89]. Remarkably, other lncRNAs have been documented to be present in both cellular compartments (the nucleus and cytoplasm), where these transcripts have versatile roles in shaping the epigenome and influencing pertinent biological processes such as transcription and translation [90,91]. Notably, recent reports are beginning to demonstrate that lncRNAs can also be present in the mitochondria [92]—alluding to the unique diversification of these RNA molecules.

In addition to their subcellular localization, the site of biogenesis can further classify lncRNAs. For example, recent updates in the classification system broadly categorize lncRNAs as either intergenic (not intersecting with any protein-coding genes) or intragenic/genic (overlapping protein-coding genes) [93,94,95]. In particular, long intergenic ncRNAs (lincRNAs) arise from intergenic regions (a span of DNA sequences situated between two genes), and albeit variably, possess a greater degree of evolutionary conservation at the sequence and RNA secondary structure level compared to intragenic lncRNAs [93,94,95,96]. Conversely, intragenic lncRNAs are transcribed in distinct regions that intersect with protein-coding loci and depending on their genomic location, these lncRNAs can be further defined as bidirectional (transcribed in a divergent manner from the promoter of a protein-coding gene on the opposite strand), intronic (originates from only the intronic regions of a protein-coding gene in either direction), antisense (transcribed on the anti-sense/non-coding strand of a protein-coding gene and may overlap the coding exons of the gene), and sense (transcribed from the sense/coding strand of the protein-coding gene and may overlap the coding exons of the gene) [93,94,95]. Despite the numerous transcriptional orientations existing for lncRNAs, it is likely that many of these RNA molecules share comparable mechanistic and functional properties that are involved in governing the genomic landscape, whether through *cis* (nearby) or *trans* (distant)-acting mechanisms.

Nevertheless, although thousands of lncRNAs continue to be annotated on a daily basis, only a small subset of these RNA molecules is functionally characterized. In comparison to their miRNA counterparts, a lot less is known about the mechanistic abilities of lncRNAs in certain disease contexts, particularly in diabetes. Therefore, in this review, we will first present the putative roles of lncRNAs in DR and inflammation, and then discuss their implications in other epigenetic mechanisms that may also contribute to the progression of inflammation in DR. Of note, we understand that the lncRNAs we will be examining in this review are non-exhaustive; however, we hope that by acknowledging the interconnectedness between these key players in inflammation and other epigenetic mechanisms, we will be able to promote novel exploratory studies that will help better understand the intricacies behind this coordinated molecular network.

## 4. LncRNAs and DR

Recent studies have made it evident that lncRNAs are dysregulated during DR [97,98,99]. As a matter of fact, in a study by Yan et al., microarray analyses of retinal tissues from 2-month old (streptozotocin-induced) diabetic mice demonstrated differential expressions of lncRNAs when compared to non-diabetic retinas; in particular, 89 lncRNAs were upregulated and 214 lncRNAs were downregulated during early DR [98]. To better understand the interactive capabilities of lncRNAs in DR, Yan et al. constructed a co-expression network between lncRNAs and mRNAs using their microarray findings and bioinformatics tools; this network comprised of 100 mRNAs and 79 differentially expressed lncRNAs that collectively contributed to 2675 network nodes. Gene ontology (GO) analyses further revealed that this regulatory network was implicated in a number of biological processes, which included cellular stress and DNA damage responses, epithelium and tube development, and tube morphogenesis. In addition to GO, the Kyoto Encyclopedia of Genes and Genomes (KEGG) pathway analyses also indicated that this co-expression network is connected to a number of signaling pathways (i.e., MAPK, chemokine signalling, pyruvate metabolism, and complement and coagulation cascades) that are involved in the progression of DR (i.e., inflammation and neovascularization). Moreover, in a separate study by Wang et al., fibrovascular membranes were obtained, through pars plana vitrectomy, from PDR patients who did or did not receive intravitreal pre-treatment with conbercept (an anti-VEGF drug), and these membranes were then subjected to RNA extraction and microarray profiling [99]. The microarray results indicated that nearly 427 lncRNAs (263 upregulated and 164 downregulated) and 571 mRNAs (192 upregulated and 379 downregulated) were differentially expressed between the two PDR patient groups. Furthermore, following the construction of a lncRNA—mRNA co-expression network, GO and KEGG analyses revealed that several of the dysregulated lncRNAs and mRNAs were involved in numerous pathways, including inflammatory signaling (i.e., TNF-α, IL-17, and nucleotide-binding and oligomerization domain (NOD)-like receptors), HIF-1 signalling, membrane trafficking (interactions with SNARE proteins) and various metabolic processes such as gluconeogenesis. Nevertheless, the findings from both studies suggest that lncRNAs are capable of participating in a variety of biological processes and any changes in the cellular milieu, whether through acute or chronic stimulus such as diabetes, can have profound effects on the global molecular network.

To further emphasize that the dysregulation of lncRNAs plays a critical role in DR, researchers performed a three-stage genome-wide association study (GWAS) involving type II diabetic Japanese patients with DR (*N* = 837) and without DR (*N* = 1149) [100]. Interestingly, after using a meta-analysis model to combine select single nucleotide polymorphisms (SNPs) from all three stages, the SNP rs9362054 was found to be strongly associated with DR. More specifically, rs9362054 is situated in an intron of the *RP1-90L14.1* gene, which encodes a lincRNA (*lnc-KIAA1009-1*) and is located between two protein-coding genes (*KIAA1009* and *TBX18*) on chromosome 6. Due to the distinct genomic localization of *RP1-90L14.1* and the fact that *KIAA1009* (also known as CEP162 and QN1) is vital for ciliogenesis [101], the investigators suspect that the *lnc-KIAA1009-1* may interconnect these genes via cis-regulation and contribute to defective ciliogenesis; raising the possibility that aberrations in key lncRNAs may facilitate the pathogenesis of certain diseases. Although additional loss-of-function and gain-of-function experiments are required to validate this hypothesis, these unique findings raise new questions regarding the molecular basis of lncRNAs in DR. In the following sections, we will take an in-depth look at some of the well-characterized lncRNAs in DR with respect to their regulatory roles in inflammation (summarized in Table 1).

## 5. LncRNAs as Novel Regulators of Inflammation in DR

### 5.1. MALAT1

Metastasis-associated lung adenocarcinoma transcript 1 (*MALAT1*) is one of the earliest lncRNAs to be identified in DR. Originally discovered in non-small cell lung carcinoma (NSCLC) [115], this highly studied intergenic lncRNA has been implicated in various cancers [116,117,118], cardiovascular disease [119], neurological disorders [120,121], skeletal myogenesis [122], and neural development [123]. Considering the ubiquitous expressions of *MALAT1* in tissues and its high degree of evolutionary conservation [124], further exploration of *MALAT1*, in particular disease contexts, will provide useful insights into the interactions between lncRNAs and the genome. Indeed, in recent years, the importance of *MALAT1* is being recognized by researchers in the field of diabetes and several functional studies are being carried out that specifically examine the dynamics of this lncRNA in diabetic complications [125,126,127]. Particularly, in DR, Yan et al. were the first to document significant aberrations in the expression of *MALAT1* in the retinas of STZ-induced diabetic mice (a type I diabetes model), RF/6A (choroid-retinal endothelial) cells cultured in high glucose, and aqueous humors and fibrovascular membranes of type II diabetic patients [98]. In addition to their in vitro and in vivo observations, *in silico* analysis further revealed that the *MALAT1* sequence contains transcription factor binding sites for NF-κB, which is a critical mediator of immune and inflammatory responses [98]. Moreover, in a separate study by the same group, elevated *MALAT1* expressions were also evident in the retinas of STZ-induced diabetic rats and db/db mice (a type II diabetes model) [106]. Additionally, several vasoactive and inflammatory markers accompanied the elevated expressions of *MALAT1* in the diabetic retina, which included VEGF, PEDF, ICAM, and TNF-α—suggesting a potential pathogenetic association for *MALAT1* in DR. Interestingly, intraocular injections of a *MALAT1* short hairpin RNA (shRNA) in the diabetic rats significantly alleviated diabetes-induced retinal inflammation, retinal cell apoptosis, vascular leakage, and electroretinogram abnormalities. Similarly, small interfering RNA (siRNA)-mediated knockdown of *MALAT1* in RF/6A cells treated with exogenous VEGF or TNF-α significantly diminished the migration and tube formation potential of endothelial cells compared to scrambled controls. The researchers in the study further determined that the MAPK pathway is critically implicated in *MALAT1′*s ability to augment proliferation in RF/6A cells during hyperglycemic stress. Specifically, western blot analyses demonstrated that *MALAT1* knockdown in HG-treated retinal cells could directly reduce the expressions of phosphorylated p38 levels; however, this knockdown did not have any visible reductions on phosphorylated JNK1/2 or ERK1/2 proteins. In order to confirm their observations, Liu et al. overexpressed *MALAT1* in RF/6A cells and then subsequently treated these cells with either chemical inhibitors or siRNAs for p38, JNK, and ERK for 48 h [106]. As expected, the p38 chemical inhibitor (SB203580) or siRNA was able to significantly impede the proliferative potential of *MALAT1*, whereas the JNK and ERK-specific treatments were unable to hinder *MALAT1*-induced hyper-proliferation—ultimately suggesting that *MALAT1* can exert its proliferative capabilities specifically through the p38 MAPK signaling pathway. Since p38 MAPK has been previously implicated in diabetes-induced retinal inflammation [128], the collective findings by Liu et al. demonstrate that other novel epigenetic molecules, such as lncRNAs, can play critical regulatory roles in the inflammatory pathways involved in the progression of DR.

To further extend the inflammatory functionalities of *MALAT1*, a recent study from our laboratory found that *MALAT1* is capable of epigenetically regulating a number of inflammatory cytokines in DR: IL-6, IL-1β, MCP-1, and TNF-α [107]. While the precise epigenetic mechanisms will be discussed in more detail in Section 6, our initial in vitro experiments determined that *MALAT1* silencing, via siRNA transfection, in human retinal endothelial cells (HRECs) significantly alleviated high glucose-induced upregulations of inflammatory cytokines. Corresponding to our observed in vitro patterns, the genetic ablation of *Malat1* (i.e., a global knockout) also diminished diabetes-induced vascular leakage and inflammation in the retinal tissues of *Malat1* knockout diabetic mice compared to wild-type diabetic controls. Similarly, in the vitreous humors of PDR patients, *MALAT1* transcript levels were significantly upregulated and pathogenetically associated with two other pro-inflammatory cytokines, IL-6 and TNF-α. Further supporting the mechanistic relationship between *MALAT1* and these inflammatory cytokines, a previous study by us demonstrated that *MALAT1* is capable of mediating IL-6 and TNF-α through the activation of its inflammatory ligand, known as serum amyloid antigen 3 (SAA3), in large vessel endothelial cells during hyperglycemia [129]. Taken together, our data indicates that the heightened production of MALAT1 promotes an inflammatory phenotype in diabetes. Aside from inflammation, it is important to note that *MALAT1* has also been shown to regulate angiogenesis in the neonatal retina [130] and in high glucose-treated HRECs [131]; after all, angiogenesis and inflammation are dynamic processes that are both actively involved in DR [132].

### 5.2. MIAT

Myocardial infarction-associated transcript (*MIAT*; also referred to as *RNCR2*, *Gomafu*, or *AK028326*) was originally identified in a case-control GWAS, where 6 SNPs in the *MIAT* locus conferred susceptibility to myocardial infarction (MI) [133]. Following this initial study, several experimental studies have emerged that shed light on the functional roles of *MIAT* in various biological and pathological processes, including schizophrenia [134], NSCLC [135], retinal and brain development [136,137], cataract formation [138], diabetic cardiomyopathy [139], and diabetic nephropathy [140]. In the context of DR, *MIAT* is significantly upregulated in the retinas of STZ-induced diabetic rats and db/db mice, and in the fibrovascular membranes of diabetic patients compared to non-diabetic controls [111]. Similar to the patterns observed in their in vivo experiments, Yan et al. also observed upregulated expressions of *MIAT* in several HG-treated retinal cell lines (i.e., RF/6A, microvascular endothelial cells, Müller cells and retinal ganglion cells) in vitro [111]. Interestingly, HG-induced upregulations of *MIAT* were also evident in two other non-retinal endothelial cell lines (HUVECs and EA.hy.926), which suggests that *MIAT* may be critically involved in endothelial cell functions during hyperglycemic stress. Furthermore, intravitreal injections of *MIAT* shRNA in the diabetic rats diminished diabetes-induced electroretinogram abnormalities, apoptosis of retinal cells and pericytes, and retinal vascular leakage. Additionally, retinal inflammation in the diabetic retinas was significantly alleviated after the knockdown of *MIAT*; in particular, Western blots demonstrated that *MIAT* shRNA is capable of downregulating TNF-α, VEGF, and ICAM proteins when compared to diabetic controls.

A recent study by Zhang et al. suggests that NF-κB and *MIAT* may share an intricate mechanistic relationship under hyperglycemic environments [112]. In fact, chromatin immunoprecipitation (ChIP) assays revealed that NF-κB (p65, the pertinent subunit of NF-κB) selectively binds to the promoter of *MIAT* and high glucose stimulation of primary rat retinal Müller cells subsequently heightens the binding activation of NF-κB with *MIAT*, compared to normal glucose controls. Moreover, pre-treatment of rat retinal Müller cells with an IKK inhibitor (Bay 11-7082) significantly downregulated the HG-induced expression levels of *MIAT*, suggesting that NF-κB may directly facilitate the regulation of *MIAT* under cellular stress. The researchers further examined the effects of *MIAT* knockdown on cultured Müller cells and observed that *MIAT* can also directly regulate HG-induced apoptosis by inhibiting the expressions and functions of miR-29b, ultimately allowing increases in the transcription factor Sp1 (lncRNA-miRNA interactions will be briefly discussed in Section 6.3) [112]. Nevertheless, the overall findings for *MIAT* strongly demonstrate that lncRNAs are implicated in several cellular networks and further research into their contributions in each network will provide new functional and mechanistic insights behind these molecules under select cellular stress responses.

### 5.3. ANRIL

Consisting of 19 exons and spanning nearly 126 kilobases (kb) [141], the antisense RNA to INK4 locus (*ANRIL*; also known as *CDKN2B-AS1*) gene gives rise to a 3.8-kb lncRNA that is prominently deregulated in cardiovascular disease [142], several cancers [143], diabetic nephropathy [144], diabetic cardiomyopathy [144], and primary open-angle glaucoma [145]. Not only is *ANRIL* deregulated in several pathologies, *ANRIL* also shares a close connection with inflammation. For example, as evidenced by Zhou et al., several isoforms of *ANRIL* are markedly upregulated in large vessel endothelial cells (HUVECs) following TNF-α treatments [146]. Additional experiments from this study determined that a putative NF-κB binding site exists in the *ANRIL* promoter sequence and TNF-α treatment is capable of augmenting the binding between NF-κB and the *ANRIL* promoter. To further support this direct binding relationship, *p65* was silenced via siRNAs and it was evident that the suppression of NF-κB impeded the TNF-α-induced *ANRIL* expressions—suggesting that NF-κB can mediate the transcriptional activity of the *ANRIL* gene. Interestingly, more downstream of NF-κB signalling, *ANRIL* is capable of regulating IL-6 and IL-8 expressions in TNF-α-treated HUVECs by directly interacting with YY1 (a RNA-binding protein and transcription factor that binds to the promoter loci of several proinflammatory genes). Namely, the authors observed that silencing *ANRIL* led to reduced YY1 binding with its *IL-6* and *IL-8* promoters, which ultimately suppressed the TNF-α-induced upregulations of IL-6 and IL-8 at both RNA and protein levels [146]. Despite the significance of *ANRIL*’s link with inflammation in large vessel endothelial cells, whether *ANRIL* exerts similar functional and mechanistic capabilities in inflammation during DR remains to be determined. However, a recent study, demonstrated by our laboratory, alludes to the angiogenic capabilities of this lncRNA in advancing DR, which becomes critical for discussion. As evident by the findings from both in vitro and in vivo experiments, hyperglycemia can significantly induce the upregulation of *ANRIL* in HG-treated HRECs and in the retinas of STZ-induced diabetic mice [97]. Not only is *ANRIL* heightened in such hyperglycemic environments, but blocking the expressions of *ANRIL* greatly hampers glucose-induced retinal angiogenesis. In particular, suppressing the expressions of *ANRIL*, via siRNAs, dramatically reduces high glucose-induced increases in endothelial cell tube formation, cellular proliferation, and VEGF RNA and protein expressions in HRECs. While, the retinal tissues from *ANRIL* knockout diabetic mice exhibited dramatic reductions in VEGF mRNA and protein levels, and retinal microvascular permeability compared to wild-type diabetic retinas. Furthermore, mechanistically, *ANRIL* was shown to govern VEGF expressions through its possible interactions with important epigenetic mediators, such as histone methylation (polycomb repressive complex 2; PRC2) and acetylation enzymes (p300), and miR-200b, during DR (these interactions will be elaborated further in Section 6.2 below).

### 5.4. H19

*H19*, a conserved and paternally imprinted lncRNA, is one of the earliest identified lncRNAs [147]. Aside from its involvement in a number of cancers [148,149,150], emerging evidence in recent years demonstrates that *H19* can influence several other pathophysiological processes such as preeclampsia [151], neural inflammation/stroke [152,153], seizure-induced brain injury [154], and corneal neovascularization [155]. *H19* can also impart its inflammatory capabilities in atherosclerosis [156]. Notably, overexpressing *H19* in HUVECs and vascular smooth muscle cells (VSMCs) restricts apoptosis and promotes the proliferative and migratory potential of both cells by upregulating the expressions of p38 and p65 (critical factors in the MAPK and NF-κB pathways, respectively) [156]. Similarly, *H19′*s inflammatory properties also extend into ischemic cerebral injury, where *H19* silencing can directly inhibit the levels of IL-1β and TNF-α, while increasing the production of IL-10 in the cerebral tissues and plasma of ischemic mice [152]. While the inflammatory-mediated mechanisms are evident for *H19* in atherosclerosis and neuroinflammation, the influence of H19 on inflammation during the progression of DR is not known. Despite the absence of literature that document this particular relationship in DR, our laboratory recently confirmed a role for *H19* in mediating the glucose-induced phenotypic switch (also known as endothelial-to-mesenchymal transition; EndMT) of endothelial cells in the diabetic retina [104]. In fact, since HG promotes the upregulation of mesenchymal markers (i.e., FSP-1, SM22, and α-SMA) and the downregulation of *H19* and endothelial cell markers (i.e., CD-31 and VE-CAD), the overexpression of *H19* in HG-treated HRECs dramatically reversed the trends evoked by hyperglycemia, which is suggestive of a protective role for *H19* in preventing EndMT in DR. Further confirming our in vitro findings, *H19* RNA levels were significantly reduced in the vitreous humors of PDR patients, and *H19* knockout control mice exhibited an EndMT retinal phenotype that were comparable to wild-type and *H19* knockout diabetic retinas. Moreover, using additional in vitro experiments, we mechanistically demonstrated that *H19* mediates EndMT through its regulation of the MAPK-ERK1/2 pathway via Smad-independent TGF-β signaling. Nevertheless, the discussion of this initial study on *H19* provides novel insights into the pathogenesis of DR and further research is warranted to explore *H19′*s implications in other DR-related molecular pathways. After all, the presence of altered extracellular matrix proteins, neovascularization, and inflammation are critical processes that collectively contribute to fibrosis and retinal tissue damage, and subsequent ocular complications [157,158].

### 5.5. BDNF-AS

The low levels of nerve growth factor BDNF (brain-derived neurotrophic factor) is linked to several neurodegenerative disorders [159]. Additionally, BDNF is implicated in the retina, where this neurotrophin is capable of promoting cellular differentiation and exerting anti-inflammatory effects in LPS-stimulated retinal pigment epithelial cells [160,161]. In the context of DR, previous studies have confirmed that the levels of BDNF are significantly reduced in the vitreous, serum, and plasma of PDR patients [161,162], while downregulated BDNF protein levels are also evident in the serum and retinal tissues of STZ-induced diabetic rats [162]. Since it is apparent that BDNF is decreased in diabetic environments, one plausible mechanism for this downregulation may be mediated by *BDNF-AS*, the natural antisense lncRNA of BDNF. In a study by Xu et al., oxygen and glucose-deprived primary retinal ganglion cells (RGCs) exhibited an inverse relationship between BDNF and *BDNF-AS* expression levels: Increases in *BDNF-AS* and reductions in BDNF [102]. This regulatory relationship was then confirmed by luciferase assays, where it was observed that *BDNF-AS* directly targets the complementary sequences of the BDNF mRNA, ultimately inhibiting the expression of this neurotrophin. Furthermore, using transduction approaches, shRNA-mediated knockdown of *BDNF-AS* in RGCs prevented ischemia-induced increases in cell apoptosis and TNF-α expressions—signifying that *BDNF-AS* plays an important role in augmenting ischemic injury. In accordance with these findings, Li et al. also demonstrate a similar phenomenon between *BDNF-AS* and BDNF expression levels in human retinal pigment epithelial cells (ARPE-19) cultured in high glucose conditions [103]. Accordingly, siRNA-mediated knockdown of *BDNF-AS* in ARPE-19 cells alleviated glucose-induced elevations in cell apoptosis and reductions in BDNF levels; thereby, conferring protection to RGCs against diabetes-associated damage. 

### 5.6. MEG3

The maternally expressed gene 3 (*MEG3*) is a lncRNA gene that belongs to the *DLK1*—*MEG3* imprinting locus and exerts critical developmental properties [163]. Several lines of evidence also suggest that the inactivation of this gene and the subsequent loss of the *MEG3* lncRNA are frequently documented in numerous cancers, suggesting important tumour-suppressive properties of this gene [164]. In diabetic environments, similar reductions of *MEG3* have been reported in both diabetic animal and in vitro models [108,109,110]. Notably, reduced serum levels of MEG3 were observed in patients with DR compared to controls; whereas, overexpression of *MEG3* in ARPE-19 cells markedly downregulated HG-induced increases of VEGF and TGF-β1 at both mRNA and protein levels [108]. Furthermore, Tong et al. report that *MEG3* overexpression in ARPE-19 cells significantly alleviates glucose-induced apoptosis and upregulations of IL-6, IL-1β, and TNF-α [109]. Following additional mechanistic experiments, the researchers concluded that *MEG3* can exert its anti-inflammatory and anti-apoptotic effects through the NF-κB and Bcl-2/Bax signaling pathways by specifically targeting two important epigenetic regulators, SIRT1 and miR-34a (these interactions will be further elaborated in Section 6.3. In a separate study by Qiu et al., intravitreal injections of *MEG3* shRNA in STZ-induced diabetic mice dramatically aggravated acellular capillaries, retinal vascular leakage, and retinal inflammation (i.e., elevated expressions of TNF-α, VEGF, IL-6, IL-1, and CCL2 were observed) [110]. Additionally, mechanistic exploratory studies demonstrated that *MEG3* knockdown is capable of decreasing apoptosis and improving cell viability in HG-treated RF/6A cells, which is further mediated by *MEG3′*s involvement in PI3K/Akt signaling [110].

### 5.7. RNCR3

Retinal non-coding RNA3 (*RNCR3*) is an intergenic lncRNA that was first documented in the developing mouse retina [136]. In addition to its role in the eye, *RNCR3* is implicated in atherosclerosis [165] and in the differentiation of oligodendrocytes and neurons [166]. Alternatively, in DR, Liu et al. demonstrates that hyperglycemia upregulates *RNCR3* and the subsequent administration of intravitreal *RNCR3* shRNA greatly impedes glial cell reactivity, as well as inducing significant reductions in many cytokines, including MCP-1, TNF-α, and VEGF-A, in the retinas of diabetic mice [113]. Additionally, the knockdown of *RNCR3* evoked neuroprotective effects on diabetic retinal tissues by improving visual function and RGC survival in the diabetic mice, while conversely decreasing glucose-induced apoptosis of retinal cells. Consistent with these findings, Shan et al. also reported similar increasing patterns of *RNCR3* in HG-treated RF/6A cells, diabetic mice retinas, and in the fibrovascular membranes of diabetic patients [114]. As well, shRNA-mediated knockdown of *RNCR3*, via intravitreal injections, reduced acellular capillaries, and retinal vascular leakage in the diabetic retinas. Interestingly, although the knockdown of *RNCR3* was shown to decrease viability, migratory potential, and tube formation of HG-treated RF/6A cells in vitro, the researchers proposed that a complex cross-talk exists, involving the *RNCR3*/KLF2 (Kruppel-like factor 2)/miR-185-5p regulatory network, which facilitates the regulation of RF/6A cells.

### 5.8. HOTTIP

HOXA transcript at the distal tip (*HOTTIP*) is a newly emerging lincRNA that resides near the 5′-end of the *HOXA* locus and is actively involved in the coordination of various *HOXA* genes, which are important in embryonic development [167]. While its disease-specific functions are being annotated, recent evidences demonstrate that *HOTTIP* dysregulation is associated with many cancers [168]. Similarly, significant aberrations of *HOTTIP* are evident in the retinas of STZ-induced diabetic rats and db/db mice [105]. Indeed, using diabetic animal models, Sun and Xu demonstrate for the first time that diabetes can induce the upregulation of *HOTTIP*. To better understand the implications of this lncRNA in DR, the researchers administered an intraocular *HOTTIP* shRNA in diabetic rats and found that the knockdown of *HOTTIP* can directly attenuate diabetes-induced electroretinogram abnormalities and retinal inflammation, which was evident through reduced expressions of VEGF and ICAM-1 proteins. Furthermore, the siRNA-mediated downregulation of *HOTTIP* dramatically reduced cell viability in RF/6A cells treated with HG or hydrogen peroxide, compared to cells only treated with HG or hydrogen peroxide—indicating that *HOTTIP* can also influence the degree of cellular apoptosis under hyperglycemic or oxidative stress conditions. In addition to their in vitro findings, *HOTTIP* silencing directly decreased phosphorylated p38 protein expressions, but did not have an impact on the phosphorylation levels of ERK1/2 and JNK1/2. Conversely, *HOTTIP*-induced cellular proliferation can be prevented by the administration of an inhibitor (SB203580) or siRNA for p38 and not by inhibitors of JNK or ERK, which alludes to the dynamic relationship between the p38-MAPK signaling pathway and *HOTTIP*.

## 6. Other Epigenetic Players Involved in the Cross-Talk between lncRNAs and Inflammation: The Missing Puzzle Pieces?

As alluded to earlier, the molecular network is complex and precisely coordinated during homeostasis. In the event of chronic hyperglycemia, the activity of various genes go awry—particularly genes associated with oxidative stress and inflammation—and damaging environments are generated that can evoke long-lasting effects despite the normalization of glucose [169,170,171]. Epigenetic mechanisms, which modify the expression of genes without changing the underlying nucleotide composition, are critically implicated in diabetes [172,173,174]. Nevertheless, presently, very few studies exist that take into consideration the complex crosstalk between lncRNAs and other epigenetic mechanisms during inflammation in DR. Therefore, in the sections below, we will discuss the three major epigenetic mechanisms in relation to lncRNAs and inflammation in the diabetic retina.

### 6.1. DNA Methylation

One of the earliest discovered epigenetic mechanisms is DNA methylation [175], which involves the interactions between two opposing enzymes that facilitate the methylation status of cytosine residues in CpG dinucleotides: either through the addition (via DNA methyltransferases; DNMTs) or removal (via DNA demethylases) of methyl groups [176]. Further, genomic regions that contain a high frequency of CpG dinucleotides are referred to as ‘CpG islands’ (CGIs), which reside in the regulatory/promoter regions of genes, and the CGIs can ultimately determine the transcriptional activity of a gene based on its degree of methylation [176,177]. For example, promoter CGIs that are hypermethylated are associated with gene silencing, while conversely hypomethylation is associated with gene activation [176,177]. Indeed, in recent years, the impact of DNA methylation has been documented in DR, where previous reports suggest that hyperglycemia can evoke distinct methylation patterns in the promoters of miRNAs [178] and several DR-related genes (i.e., *MMP-9* and *TNF*) [179,180,181], furthering the progression of DR. Adding to these results, findings from our recent study demonstrate for the first time that DNA methylation is closely connected with *MALAT1* and its inflammatory mediators in DR pathogenesis [107]. In fact, blocking DNMTs (through the administration of pan-DNMT inhibitors or a *DNMT1* siRNA, which is a constitutively expressed DNMT) in HRECs cultured in NG or HG conditions further exacerbated glucose-induced RNA expressions of *MALAT1*, *IL-6*, *TNF-α*, *MCP-1*, and *IL-1β*—indicating that DNMTs actively participate in the transcriptional regulation of several genes. Moreover, using a DNA methylation array, we then closely examined the CpG sites across the *MALAT1* gene in both NG and HG-treated HRECs. Intriguingly, we observed that transient glucose treatments (48 h) did not significantly alter the methylation status of the CGI in the *MALAT1* promoter. While we conducted our DNA methylation experiment at one particular time-point, it would be intriguing to see whether initial hyperglycemic treatments can provoke persistent, long-lasting changes in the methylation status of the CGI in the *MALAT1* promoter. Constructing such an in vitro cell culture model involving multiple time-points and alternating glucose treatments will provide unique insights behind metabolic memory and the regulatory nature of DNA methylation on the biogenesis of lncRNAs during the progression of DR.

### 6.2. Histone Modifications

Another fundamental and well-studied epigenetic mechanism that is involved in the coordination of gene expression is histone modifications. Histone-modifying enzymes, such as histone methyltransferases, histone demethylases, histone acetyltransferases, and histone deacetylases, coordinate their actions by chemically modifying particular amino acid residues within the histone proteins (H2A, H2B, H3, and H4), which subsequently governs the overall conformation of the chromatin and its accessibility to transcription factors for gene transcription at that modified region [182,183,184,185,186]. For example, a euchromatin (open) configuration is induced by histone acetyltransferases through the acetylation of lysine residues, which generally leads to active gene transcription [182,183,184]; whereas, depending on the degree of methylation and specific residue, histone methyltransferases facilitate the methylation of lysine residues that can drive gene silencing (a heterochromatin state) or activation [185,186]. Changes in histone modifications have been extensively reported in multiple cancers [187] and in recent years, several studies have also documented the presence of aberrant histone modifications in diabetic environments [188,189,190,191,192,193,194]. Despite the breadth of information, very few studies have addressed the involvement of histone modifications on lncRNA-mediated mechanisms in DR. In fact, presently, only histone methylation and acetylation have been shown by our laboratory to influence lncRNAs in DR, which will be the topic of discussion in the paragraphs below.

Polycomb repressive complex 2 (PRC2) is a multimeric histone methyltransferase complex that catalyzes the tri-methylation of lysine 27 on histone 3 (H3K27me3), a distinct chromatin mark linked with gene repression [195]. A previous study from our laboratory demonstrated that the core components of PRC2 (EZH2, SUZ12, and EED) were significantly elevated in HG-treated HRECs and retinal tissues of diabetic rats and mice, which were also accompanied by increased VEGF expressions and reduced miR-200b levels (a negative regulator of VEGF) [196]. Furthermore, using ChIP-qPCR analyses to confirm the initial observations between PRC2 and miR-200b, HG-treated HRECs exhibited increased H3K27me3 and decreased RNA polymerase 2 associations in the promoter region of *miR-200b* when compared to NG controls. Interestingly, in vitro disruption of PRC2 with 3-Deazaneplanocin (DZNep) dramatically prevented HG-induced reductions of miR-200b, while VEGF RNA and protein levels were dramatically decreased in parallel—suggesting that PRC2 can negatively regulate miR-200b, while indirectly promoting the expressions of VEGF, in hyperglycemic environments. Further extending the regulatory mechanisms of PRC2, in vitro and in vivo analyses from our recent studies also revealed that lncRNAs are intimately connected with PRC2 functions in diabetes [97,107]. Beginning with *ANRIL*, retinal tissues from *ANRIL* knockout diabetic mice revealed depressed expressions of *EZH2* and *EED* RNA levels (and no changes in *SUZ12* expressions), when compared to wild-type diabetic retinas. Similar observations were additionally reported in HG-treated HRECs following *ANRIL* silencing—confirming *ANRIL*’s direct impact on the EZH2 and EED subunits of PRC2. On the other hand, administration of DZNep in HG-treated HRECs significantly reduced both *ANRIL* and *VEGF* RNA expressions, which suggests that a highly interactive network may exist between these molecules; after all, RNA immunoprecipitation analyses demonstrated that HG could promote a strong binding association between EZH2 and *ANRIL* [97]. Of note, *ANRIL* also shared a similar relationship with p300 [97], which is a prominent histone acetyltransferase involved in the regulation of several glucose-related genes [197,198,199]. Generally, HG-induced upregulations of p300 were corrected following *ANRIL* silencing in HRECs and *ANRIL* knockout diabetic mice exhibited reduced retinal *p300* levels compared to wild-type diabetic retinas. Interestingly, transfection with siP300 in HG-treated HRECs did not alter *ANRIL* expressions [97].

Nearly analogous to *ANRIL*, the reductions of *MALAT1*, through silencing or knockout strategies, significantly prevented diabetes-induced increases in *EZH2*, *SUZ12*, and *EED* RNA levels [107]. Interestingly, DZNep pre-treatment was capable of reducing glucose-induced upregulations of *MALAT1* and *TNF-α*, but conversely exacerbated the expressions of *IL-6*, *MCP-1*, and *IL-1β*; these findings may allude to the context-specific regulation of PRC2 on target gene expressions [200]. Moreover, the relationship between *MALAT1* and PRC2 was quite evident in HRECs, as strong binding associations were also observed and the in vitro silencing of *MALAT1* directly reduced EZH2 protein levels in HG environments. Collectively, these findings allude to the potential abilities of lncRNAs to form scaffolds or act as guides with certain chromatin-modifying enzymes in diabetic environments. Further mechanistic-based studies are warranted that closely examine the relationship between these key epigenetic players in mediating the pathogenesis of DR.

### 6.3. miRNAs

miRNAs (miRs) have emerged as critical post-transcriptional regulators of gene expression [201,202]. Despite being ~22 nucleotides in length, these small ncRNAs exert their powerful functions by binding to the 3′ untranslated region (3′-UTR) of their target mRNAs, which subsequently leads to mRNA degradation and/or the inhibition of protein translation [203]. MiRs have been implicated in cancers [204], cardiovascular disease [205], neurodegenerative diseases [206] and within the last decade, numerous miRs have been identified in DR [199,207,208,209,210]. For the purposes of this review, we will briefly look at few of the documented miRs that are known to interact with lncRNAs during the progression DR.

Tong et al. shed novel insights into the regulatory capabilities of *MEG3* on a molecular axis involving SIRT1 (a histone deacetylase) and miR-34a in retinal epithelial cells [109]. With HG environments promoting the upregulation of miR-34a and downregulations of *MEG3* and *SIRT1*, the subsequent overexpression of *MEG3* in ARPE-19 cells dramatically reversed the HG-induced effects—confirming the inverse relationships shared between *MEG3*, SIRT1, and miR-34a. Furthermore, incorporating miR-34a mimics and inhibitors into their in vitro experiments, the authors confirmed that miR-34a could negatively regulate *SIRT1*. *In silico* analyses and luciferase experiments were then carried out that confirmed *MEG3′*s ability to positively regulate SIRT1 by directly sponging its negative regulator, miR-34a. Additionally, *MEG3* is capable of reducing HG-induced apoptosis and inflammation by downregulating miR-34a levels. Tong et al. also indicated that either *MEG3* overexpression or miR-34a knockdown is capable of upregulating the levels of SIRT1 by reducing the HG-induced activation of the NF-κB signaling pathway.

Shan et al. also established that the lncRNA *RNCR3* is upregulated in RF/6A cells cultured in HG and associated with retinal vascular dysfunction in vivo [114]. In addition to their initial findings, the authors wanted to examine the regulatory role of miR-185-5p on *RNCR3* and KLF2 expressions in RF/6A cells, since their previous atherosclerosis-based study determined that a feedback loop existed between these molecules [165]. It was determined that miR-185-5p directly regulates *RNCR3* and KLF2 expressions, since the levels of *RNCR3* and KLF2 decreased after the administration of miR-185-5p mimics. Furthermore, the knockdown of *RNCR3* or the presence of miR-185-5p mimics both contributed to reduced cell viability and proliferation, whereas KLF2 overexpression increased cell viability and proliferation—alluding to the potential regulatory network in RF/6A cells.

Moreover, *MIAT* was shown to function as a molecular decoy/sponge that sequesters miR-29b and miR-150-5p, which subsequently promotes the expression of their target mRNAs [111,112]. In particular, Yan et al. first used bioinformatics tools to identify predicted binding sites of miR-150-5p on its target mRNA, *VEGF*, and its target lncRNA, *MIAT* [111]. Using this information, the authors then cloned the specific regions to luciferase vectors and consequently, transfected RF/6A endothelial cells with these vectors and miR-150-5p mimics. Interestingly, the luciferase assays demonstrated that miR-150-5p directly targets *VEGF* and *MIAT*. Following these findings, the authors wanted to gain a better understanding of the decoy/sponge functions of *MIAT* in vitro, so increasing levels of miR-150-5p were administered to RF/6A cells in the absence or presence of *MIAT*. It was observed that VEGF expressions were dramatically upregulated during *MIAT* overexpression, while conversely VEGF levels significantly decreased in *MIAT*-overexpressing endothelial cells with increasing levels of miR-150-5p. To determine whether similar patterns exist vice-versa, the authors also administered increasing levels of *MIAT* in the presence or absence of miR-150-5p. Indeed, the gradual increases in *MIAT* were capable of restoring the miR-150-5p-induced downregulations of VEGF in miR-150-5p-overexpressing RF/6A cells, which confirms the interplay between these molecules. This regulatory cross-talk between *MIAT*, miR-150-5p, and VEGF was also implicated in the critical functions of endothelial cells during high glucose stress [111]. Additional findings by Zhang et al. demonstrate that *MIAT* is also capable of promoting Sp1 through the suppression of its negative regulator, miR-29b, in HG-treated rat retinal Müller cells—ultimately leading to heightened levels of apoptosis [112]. In fact, *MIAT* knockdown was capable of elevating miR-29b levels and cell viability, while decreasing the increased levels of Sp1 and apoptosis in HG-treated Müller cells.

## 7. Conclusions

**Figure 1:**
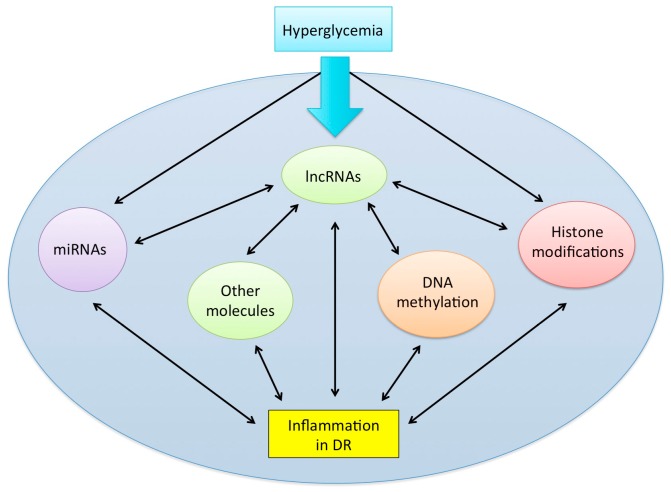
A schematic depicting the dynamic, coordinated network involving epigenetic modifications in inflammation during DR. Several key epigenetic mechanisms are involved in the progression of inflammation. LncRNAs may serve as critical regulators of inflammation, through their effects on other epigenetic mechanisms, such as DNA methylation, histone modifications, and the activity of other non-coding RNAs (i.e., miRNAs). Furthermore, since the molecular network is heavily coordinated, several individual components of this network may inter-regulate one another, indicated by the double arrows in the figure, and future research should keep these interactions in mind.

Undoubtedly, the recent emergence of lncRNAs has evolved our understanding of pathogenetic mechanisms in inflammatory-driven diseases including DR. Gain-of-function and loss-of-function experiments have also made it evident that lncRNAs are dynamic regulators of gene expression. As fields continue to annotate lncRNAs, a dire need remains for DR research that elucidate the underpinnings of the epigenome network in order to understand the driving factors that initiate inflammatory changes. Integrated analyses that take into consideration this complex molecular landscape (please see Figure 1) will bring new questions to light regarding the epigenetic paradigm and provide novel avenues for better-targeted therapeutic and diagnostic options. In this review, not only have we highlighted the inflammatory-based roles of lncRNAs in DR, but we have also attempted to address other epigenetic mechanisms that are implicated in this coordinated regulation of inflammation. While the comprehension of this network is a work in progress in the field of DR, we hope that future studies will take these considerations into account when examining the epiphenomena. Nevertheless, continuing to explore the genomic landscape and its intricacies will provide novel mechanistic insights and discussions for the functions of lncRNAs in DR. To conclude, as DR prevalence reaches epidemic levels globally, understanding the inflammatory-mediated roles of lncRNAs in DR development and progression is critical for improving the diabetic standard of care in not only diagnostic testing, but also in the management and treatment of DR.

## Figures and Tables

**Table 1:** Pertinent lncRNAs that are involved in DR.

lncRNA	Reported Functions in DR	Implications in Other Epigenetic Mechanisms
*ANRIL*	Expressions are upregulated in HG-treated HRECs and in the retinas of diabetic mice [97] siRNA-mediated knockdown of *ANRIL* significantly hampers high glucose-induced increases in endothelial cell tube formation, cellular proliferation, and VEGF RNA and protein expressions in HRECs [97] Retinal tissues from *ANRIL KO* diabetic mice exhibited dramatic reductions in VEGF mRNA and protein levels, and retinal microvascular permeability compared to wild-type diabetic retinas [97]	*ANRIL* was shown to govern VEGF expressions through its possible interactions with important epigenetic mediators, such as histone methylation (polycomb repressive complex; PRC2) and acetylation enzymes (p300), and miR-200b, during DR [97]
*BDNF-AS*	Upregulations of *BDNF-AS* are observed in oxygen and glucose-deprived primary RGCs [102] *BDNF-AS* directly targets the complementary sequences of the BDNF mRNA (a neurotrophin), ultimately inhibiting the expression of the protein [102] Knockdown of *BDNF-AS* in ARPE-19 cells alleviates glucose-induced elevations in cell apoptosis and reductions in BDNF levels [103]	*BDNF-AS* and its implications in other epigenetics mechanisms have not been documented yet in DR-based studies
*H19*	Significantly downregulated in HG-treated HRECs, vitreous humors of diabetic patients, and in the retinas of STZ-induced diabetic mice [104] Overexpression of *H19* dramatically reverses HG-induced EndMT changes in retinal endothelial cells; while, retinas from *H19* KO control mice exhibited an EndMT retinal phenotype that were comparable to wild-type and *H19* knockout diabetic retinas—suggesting a protective role for *H19* in DR [104] *H19* mediates EndMT through its regulation of the MAPK-ERK1/2 pathway via Smad-independent TGF-β signalling	*H19* overexpression is capable of rescuing the glucose-induced downregulations of miR-200b (a protective miRNA in DR) in HRECs [104] Additional in vitro experiments using miR-200b mimics and *H19* overexpression revealed that *H19* is a positive regulator of miR-200b [104]
*HOTTIP*	Significant upregulations of *HOTTIP* are evident in the retinas of STZ-induced diabetic rats and db/db mice [105] Intraocular knockdown of *HOTTIP* in diabetic rats directly attenuates diabetes-induced electroretinogram abnormalities and retinal inflammation [105] In vitro downregulation of *HOTTIP* reduces cell viability in RF/6A cells treated with HG or hydrogen peroxide [105] *HOTTIP* induces cellular proliferation through its dynamic relationship with the p38-MAPK signalling pathway [105]	*HOTTIP* and its involvement in other epigenetic mechanisms during the progression of DR have not been elucidated yet
*MALAT1*	Upregulated in diabetic animal retinas, HG-treated retinal cell lines, and in the aqueous humors, vitreous humors and fibrovascular membranes of diabetic patients [98,106,107] Knockdown of *MALAT1* in the diabetic retinas of animals can alleviate diabetes-induced retinal inflammation, retinal cell apoptosis, vascular leakage, and electroretinogram abnormalities [106] Exerts proliferative capabilities in glucose-treated retinal endothelial cells through p38 MAPK signalling [106] Promotes an inflammatory phenotype, influences diabetes-induced vascular leakage, and regulates IL-6, IL-1β, MCP-1, and TNF-α cytokines in DR [107].	Inhibition of DNA methylation can exacerbate *MALAT1* and its inflammatory mediators [107] *MALAT1* shares a strong binding association with EZH2 (the catalytic subunit of PRC2) in HG environments [107] Blocking histone methylation decreases *MALAT1* and evokes differential expressions of diabetes-related genes [107]
*MEG3*	Reductions are of *MEG3* have been reported in both diabetic animal and in vitro models [108,109,110] *MEG3* serum levels are decreased in DR patients; while, overexpression of *MEG3* in ARPE-19 cells markedly downregulates HG-induced increases of VEGF and TGF-β1 [108] Intravitreal injections of *MEG3* shRNA in STZ-induced diabetic mice dramatically aggravated acellular capillaries, retinal vascular leakage, and retinal inflammation [109]	*MEG3* can exert its anti-inflammatory and anti-apoptotic effects through the NF-κB and Bcl-2/Bax signalling pathways by specifically targeting two important epigenetic regulators, SIRT1 (a histone deacetylase) and miR-34a [109]
*MIAT*	Significantly upregulated in the retinas of diabetic animals, fibrovascular membranes of diabetic patients and retinal cell lines cultured in HG [111]. Intravitreal injections of *MIAT* shRNA in diabetic rats diminishes diabetes-induced electroretinogram abnormalities, inflammation, apoptosis of retinal cells and pericytes, and retinal vascular leakage [111] Hyperglycemic environments heightens the binding activation of NF-κB with *MIAT* in retinal Müller cells [112]	Regulatory cross-talk exists between *MIAT*, miR-150-5p, and VEGF and is implicated in the critical functions of endothelial cells during high glucose stress [111] *MIAT* can directly regulate HG-induced apoptosis by inhibiting the expressions and functions of miR-29b, ultimately allowing increases in the transcription factor Sp1 [112]
*RNCR3*	Hyperglycemia upregulates *RNCR3* in retinal cells, diabetic animal retinas, and fibrovascular membranes of diabetic patients [113,114] Administration of intravitreal *RNCR3* shRNA greatly impedes glial cell reactivity, as well as inducing significant reductions in many cytokines, including MCP-1, TNF-α, and VEGF-A, in the retinas of diabetic mice [113] Knockdown of *RNCR3* was shown to decrease viability, migratory potential, and tube formation of HG-treated RF/6A cells [114]	A complex cross-talk involving the *RNCR3*/KLF2 (Kruppel-like factor 2)/miR-185-5p regulatory network, facilitates the regulation of RF/6A cells [114] *RNCR3* knockdown or the presence of miR-185-5p mimics in RF/6A cells both contribute to reduced cell viability and proliferation, whereas KLF2 overexpression increases cell viability and proliferation [114]

STZ = streptozotocin; DR = diabetic retinopathy; HG = high glucose; NG = normal glucose; HRECs = human retinal endothelial cells; KO = knockout; RGCs = retinal ganglion cells; EndMT = endothelial-to-mesenchymal transition.
